# Gas nitriding and subsequent oxidation of Ti-6Al-4V alloys

**DOI:** 10.1186/1556-276X-7-21

**Published:** 2012-01-05

**Authors:** Dong Bok Lee, Iryna Pohrelyuk, Oleh Yaskiv, Jae Chun Lee

**Affiliations:** 1 School of Advanced Materials Science and Engineering, Sungkyunkwan University, Suwon, 440-746, South Korea; 2 Physico-Mechanical Institute of National Academy of Sciences, Lviv, 79601, Ukraine; 3 Department of Materials Science and Engineering, Myongji University, Yongin, 449-728, South Korea

**Keywords:** titanium, nitriding, nitrogen, oxidation

## Abstract

Ti-6Al-4V alloys consisting of α-Ti grains and intergranular β-Ti islands were nitrided at 850°C for 1 to 12 h under a nitrogen pressure of 1 Pa. With increasing nitriding time, the Ti-N compound layer became thicker, and the α-Ti diffusion zone containing dissolved nitrogen became wider. In the Ti-N compound layer, the initially formed Ti_2_N became TiN as the nitriding progressed. The nitride layers were oxidized to rutile-TiO_2 _after oxidation at 700°C for 10 h in air.

## Introduction

Titanium alloys are widely used in the aircraft, automobile, chemical, and biomedical industries due to their high specific strength, good corrosion resistance, and biocompatibility. However, their main drawbacks are their low hardness and poor wear resistance. In order to overcome these problems, various nitriding techniques including diffusion, ion-plasma, detonation, laser, and high-energy methods have been applied to synthesize TiN surface layers [1-7]. TiN films are the most widely used films in such industrial applications as cutting tools, die molds, mechanical parts, diffusion barriers in microelectronics, and decorative items [8-10]. In this study, the gas nitriding technique, a type of thermodiffusion treatment, was utilized to synthesize TiN films on the Ti-6Al-4V alloy. It takes advantage of the high reactivity of titanium with nitrogen to produce hardened surface layers that are well bonded to the tough matrix, without deteriorating the mechanical properties. For industrial application, a full understanding of the gas nitriding technique and high-temperature oxidation behavior of the nitrided Ti alloys is necessary because these wear-resistant, hard TiN films are frequently exposed to oxidative atmospheres during their service life. Since TiN films begin to oxidize at temperatures as low as 550°C, their thermal stability is important [11,12]. However, the effect of oxidation on nitrided Ti alloys is not well established. The diffusion of oxygen from the atmosphere to the reaction interface or the desorption of nitrogen from the reaction interface to the atmosphere was proposed as the main factor governing the oxidation of TiN films [12-14]. The purpose of this study is to investigate the nitride layers that formed on Ti-6Al-4V alloys under controlled gas nitriding conditions and their high-temperature oxidation characteristics.

## Experimental details

Ti-6Al-4V alloy was used as the substrate as it is the most widely used titanium alloy. The substrates were cut into dimensions of 15 × 10 × 1 mm^3^, polished with a 0.1-μm diamond paste to reduce the maximum value of the roughness, *R*_a_, to 0.4 μm, degreased in benzene, washed with deionized water, and nitrided via the following gas nitriding technique. The substrates were placed in the reaction chamber inside the furnace in a vacuum of 10^-3 ^Pa, heated to 850°C at a heating rate of 0.04°C/s, held at this temperature for 1, 6, or 12 h at *P*_N2 _= 1 Pa, cooled to 500°C at a cooling rate of 0.03°C/s at *P*_N2 _= 1 Pa, and further cooled to room temperature in a vacuum of *P*_N2 _= 10^-3 ^Pa. Nitrogen was deoxygenated by filtering the moisture and oxygen through silica gel and titanium chips at 1, 000°C. Oxidation tests on the nitrided specimens were conducted at 700°C in atmospheric air for 10 h.

The nitrided and subsequently oxidized specimens were investigated by scanning electron microscopy [SEM], electron probe microanalysis [EPMA], X-ray diffraction [XRD] with CuKα radiation at 40 kV and 300 mA, and transmission electron microscopy [TEM] (operated at 200 keV) in conjunction with EDS with a 5-nm spot size. The TEM sample was prepared by milling in a focused ion beam system after carbon coating.

## Results and discussion

**Figure 1:**
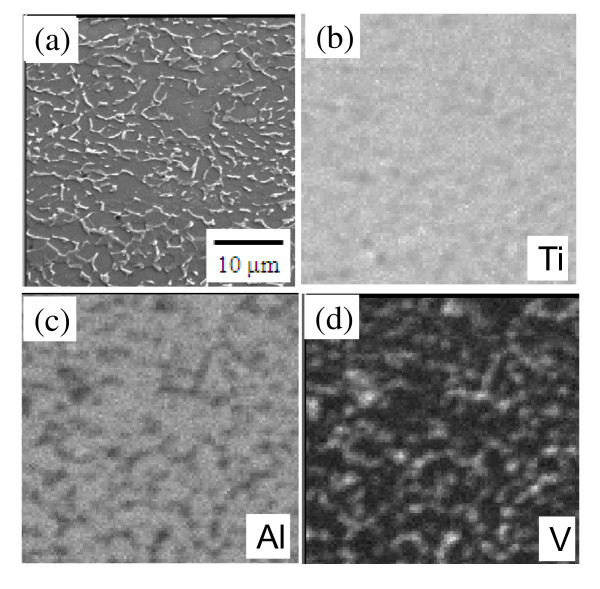
**Ti-6Al-4V alloy before nitriding (etched with Kroll's etchant; 2% HF in water)**. (**a**) EPMA image. Maps of (**b) **Ti, (**c**) Al, and (**d**) V.

Figure 1 shows the EPMA image and the corresponding elemental maps of the Ti-4Al-6V substrate. The α-Ti grains (dark area in Figure 1a) were rich in Al, and the intergranular β-Ti (white islands in Figure 1a) was rich in V. This is due to the fact that Al is an α stabilizer, while V is a β stabilizer.

**Figure 2:**
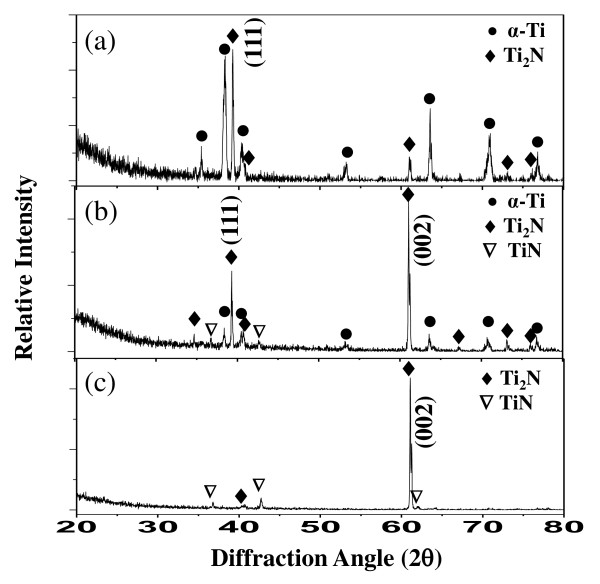
**XRD patterns of Ti-6Al-4V alloys**. Nitriding at 850°C for (**a**) 1, (**b**) 6, and (**c**) 12 h.

Figure 2 shows the XRD patterns of the Ti-4Al-6V alloys nitrided at 850°C. After nitriding for 1 h, a distinct, tetragonal Ti_2_N layer was formed on the α-Ti-rich matrix (Figure 2a). After nitriding for 6 h, the α-Ti-rich matrix peaks became weaker and weak fcc-TiN peaks appeared, owing to the increased nitriding time (Figure 2b). Here, Ti_2_N began to exhibit a preferred orientation along the (002) direction. After nitriding for 12 h, the α-Ti matrix peaks disappeared, whereas weak TiN peaks and strong Ti_2_N peaks with a (002) preferred orientation appeared (Figure 2c). Hence, it is seen that the α-Ti-rich matrix transformed into Ti_2_N from the surface and later, into TiN as the nitriding progressed. Ti_2_N exists in a narrow range of approximately 34 at.%N, while TiN displays a wide range of nitrogen solubility above 38 at.%N at 850°C in the Ti-N phase diagram. The formation of Ti_2_N indicates that the minimum nitrogen content of 34 at.%N is attained after nitriding for 1 h (Figure 2a). The Ti-6Al-4V alloy exhibits an allotropic transition between the low-temperature hcp α-Ti and the high-temperature bcc β-Ti at 995°C. Since nitriding was performed at a temperature lower than the β-transus temperature, α-Ti, TiN, and Ti_2_N were detected in Figure 2a, b. When the Ti alloys were nitrided at 950°C to 1, 050°C for 1 to 5 h in atmospheric nitrogen, surface layers of TiO_2_, TiN, Ti_2_N, and α-Ti with N in a solid solution (viz. α-Ti(N)) were formed [1-3]. In this study, the residual oxygen was well regulated so as not to form TiO_2_.

**Figure 3:**
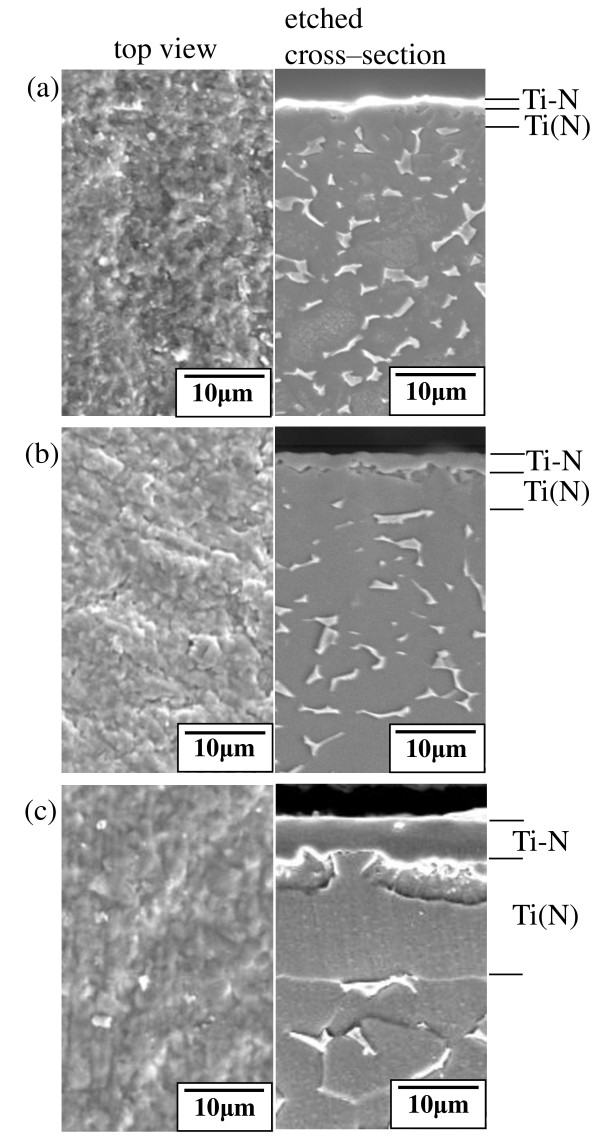
**SEM top-view and etched cross-sectional images of Ti-6Al-4V alloys**. Nitriding at 850°C for (**a**) 1, (**b**) 6, and (**c**) 12 h. Ti-N, compound layer; Ti(N), diffusion zone.

Figure 3 shows the SEM images of the nitrided Ti-4Al-6V alloys. Regardless of the nitriding time, all of the surfaces exhibited a golden yellow color and consisted of fine nitrides with a smooth surface. The nitrided Ti-4Al-6V alloys in all of the cross-sectional images consisted of an outer Ti-N compound layer, inner α-Ti(N) diffusion zone, and matrix. According to Figure 2, the compound layer consisted of Ti_2_N with and without TiN, and the diffusion zone consisted of α-Ti having dissolved nitrogen. It is noted that α-Ti can dissolve up to 7.6 wt.% nitrogen. After nitriding for 1 h, the thicknesses of the Ti-N layer and Ti(N) zone were 0.8 and 2.5 μm, respectively (Figure 3a). After nitriding for 6 h, their thicknesses were 2.3 and 4.6 μm, respectively (Figure 3b), and after 12 h, their thicknesses were 5 and 14 μm, respectively (Figure 3c). On the other hand, when pure Ti was nitrided at 1, 100°C for 12 h in atmospheric nitrogen, a 20-μm-thick TiN layer and a 50-μm-thick α-Ti(N) layer were formed [4]. Also, when pure Ti was nitrided at 1, 250°C for 5 h in atmospheric nitrogen, a 35-μm-thick TiN layer was formed [6]. The thicknesses of the nitride layers were larger, and Ti_2_N was not detected in Vojtěch et al. [4] and Seahjani and Cimenoglu [6]. This discrepancy from the results obtained in this study is attributed to the higher nitriding temperatures and pressures employed in Vojtěch et al. [4] and Seahjani and Cimenoglu [6]. Intergranular β-Ti islands within the Ti(N) zone were not recognizable in the cross-sectional images because nitrogen is a potent α stabilizer, and the diffused nitrogen transformed the intergranular β into α. As the nitriding time increased, the thickness of the Ti-N layer and moreover, that of the Ti(N) zone increased together with the grain growth of the matrix.

**Figure 4:**
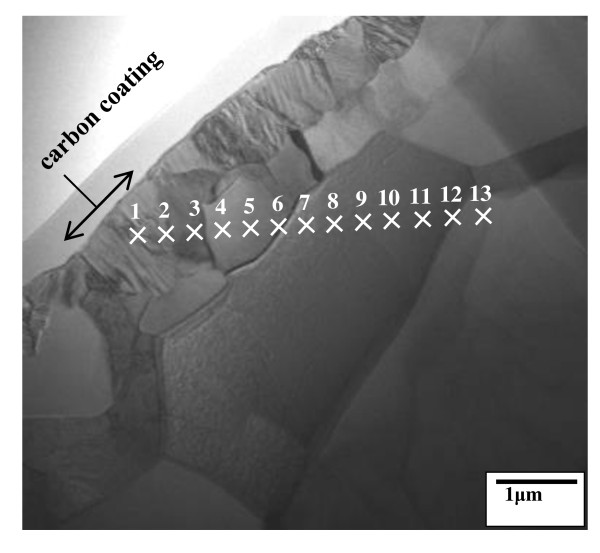
**TEM cross-sectional bright-field image of the Ti-6Al-4V alloy after nitriding at 850°C for 1 h**.

Figure 4 shows the TEM cross-sectional image of the Ti-6Al-4V alloy after nitriding at 850°C for 1 h. The elemental concentrations along spots 1 to 13 are listed in Table 1. It is however noted that the N-Kα, Ti-Lα, and V-Lα spectra overlap at approximately 0.39 keV, and the signal of nitrogen with a low atomic number is attenuated because of its low characteristic energy. Hence, the concentrations listed in Table 1 are tentative. Nevertheless, nitrogen diffused interstitially to form an outer, 0.9-μm-thick Ti-N layer (spots 1 and 2). There should exist an inner Ti(N) zone below spot 3. Aluminum was locally segregated at spots 3 to 6 due to its limited solubility in the nitrides [7].

**Table 1:** Concentration of spots 1 to 13 shown in Figure 4 (at.%)

Spot number	Ti	Al	V	N
1	90.2	0	0	9.8
2	85.5	0	0	14.5
3	79.7	18.6	1.7	0
4	77.6	20.9	1.5	0
5	80.5	18.1	1.4	0
6	85.5	12.4	2.1	0
7	87.3	11.7	1.0	0
8	87.8	10.9	1.3	0
9	88.4	10.3	1.3	0
10	88.8	10.1	1.1	0
11	88.8	9.8	1.4	0
12	88.4	10.2	1.4	0
13	88.8	9.7	1.5	0

**Figure 5:**
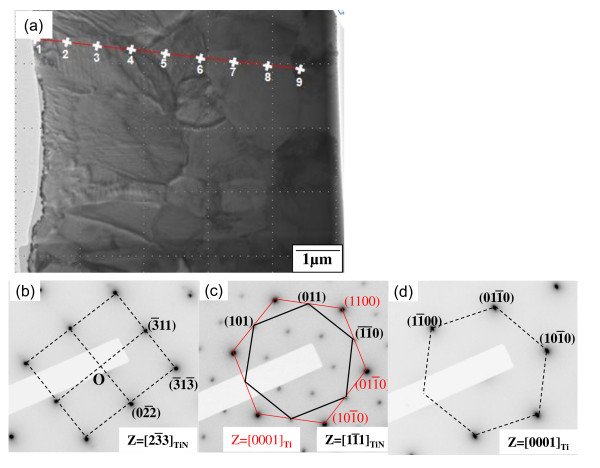
**Ti-6Al-4V alloy after nitriding at 850°C for 12 h**. (**a**) TEM cross-sectional bright-field image. Selected area electron diffraction patterns of spots (**b**) 3, (**c**) 5, and (**d**) 7.

Figure 5a shows the cross-sectional image of the Ti-6Al-4V alloy after nitriding at 850°C for 12 h. The elemental concentrations along spots 1 to 9 are listed in Table 2. Spots 1 to 6 correspond to the outer, 4-μm-thick Ti-N layer, below which the inner Ti(N) zone exists. Spots 3, 5, and 7 were determined to be TiN (Figure 5b), TiN plus α-Ti(N) (Figure 5c), and α-Ti(N) (Figure 5d), respectively. Aluminum tended to be depleted around the outer Ti-N layer and be enriched at spots 7 and 8. Such a tendency was however weak for vanadium.

**Table 2:** Concentration of spots 1 to 9 shown in Figure 5 (at.%)

Spot number	Ti	Al	V	N
1	56.0	1.2	5.3	37.5
2	91.8	0	2.8	5.4
3	89.2	0	2.9	7.9
4	89.5	0.7	3.3	6.5
5	78.4	5.3	6.5	9.8
6	88.0	5.1	5.0	1.9
7	85.1	9.4	5.4	0.1
8	85.0	8.5	6.5	0
9	86.8	6.3	6.9	0

**Figure 6:**
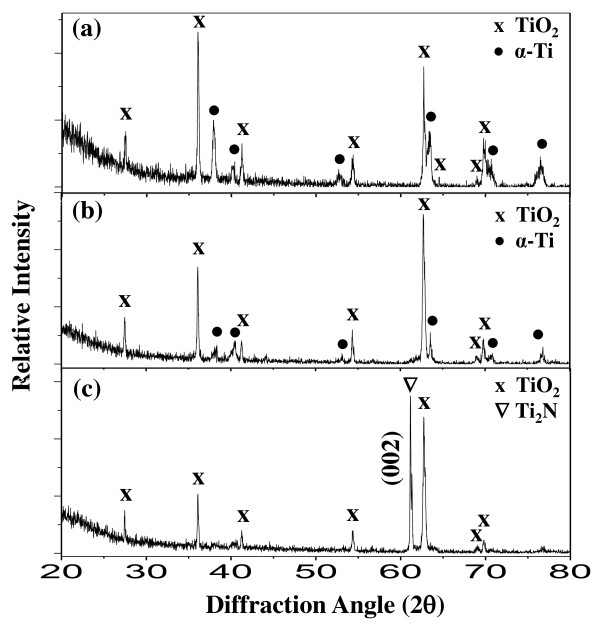
**XRD patterns of Ti-6Al-4V alloys**. Nitriding at 850°C for (**a**) 1, (**b**) 6, and (**c**) 12 h in nitrogen gas, followed by oxidizing at 700°C for 10 h in air.

Figure 6 shows the XRD patterns of the Ti-4Al-6V alloys after nitriding at 850°C for 1, 6, or 12 h, followed by oxidization at 700°C for 10 h in air. For the samples nitrided for 1 and 6 h, the initial TiN and Ti_2_N nitrides were completely oxidized to rutile-TiO_2 _on the α-Ti matrix. In the case of the sample nitrided for 12 h, Ti_2_N with a preferred orientation along (002) was still retained underneath the TiO_2 _surface scale. It is noted that Ti nitrides have better oxidation resistance than α-Ti because of the strong interaction between titanium and nitrogen, which decreases the thermodynamic activity of titanium and acts as a diffusion barrier against the inward diffusion of oxygen [5].

**Figure 7:**
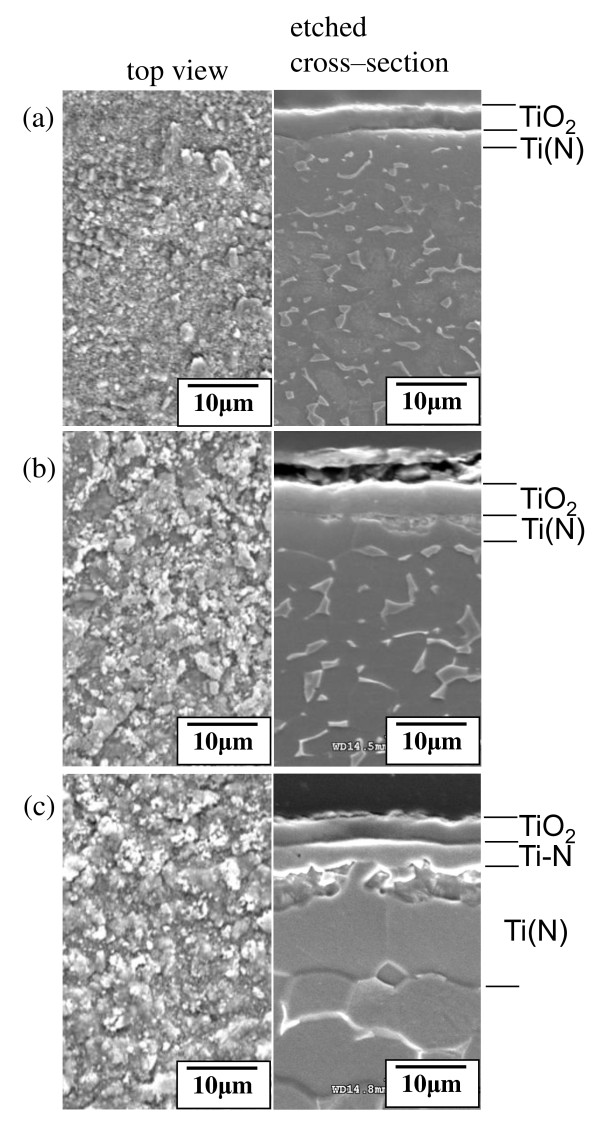
**SEM top-view and cross-sectional images of Ti-6Al-4V alloys**. Nitriding at 850°C for (**a**) 1, (**b**) 6, and (**c**) 12 h in nitrogen gas, followed by oxidizing at 700°C for 10 h in air.

The samples outlined in Figure 6 were inspected by SEM as shown in Figure 7. All top views display fine oxide grains with rather rough surfaces. All of the cross-sectional images indicate the presence of 3- to 4-μm-thick oxide layers. In Figure 7a, b, the Ti-N layers with original thicknesses of 0.8 to 2.3 μm were completely oxidized. In Figure 7c, the Ti-N layer with an original thickness of 5 μm was partially oxidized.

## Conclusions

The nitriding of Ti-6Al-4V alloys at 850°C at *P*_N2 _= 1 Pa resulted in the dissolution of the interstitial nitrogen and the formation of nitrides. When nitrided for 1 h, a 0.8-μm-thick Ti-N compound layer that consisted of Ti_2_N and a 2.5-μm-thick α-Ti(N) diffusion zone that consisted of Ti having dissolved nitrogen were formed. When nitrided for 6 h, a 2.3-μm-thick compound layer consisting of Ti_2_N and TiN and a 4.6-μm-thick α-Ti(N) diffusion zone were formed. When nitrided for 12 h, a 5-μm-thick compound layer consisting of Ti_2_N and TiN and a 14-μm-thick α-Ti(N) diffusion zone were formed. Aluminum tended to be depleted at the Ti-N compound layer. The nitrides that were formed were oxidized to rutile-TiO_2 _during oxidation at 700°C in air.

## Competing interests

The authors declare that they have no competing interests.

## Authors' contributions

DBL carried out the oxidation studies and wrote the manuscript. IP and OY performed the gas nitriding. JCL participated in the design of the study and performed the structural studies. All authors read and approved the final manuscript.
